# Cold Atmospheric Plasma Stimulates Clathrin-Dependent Endocytosis to Repair Oxidised Membrane and Enhance Uptake of Nanomaterial in Glioblastoma Multiforme Cells

**DOI:** 10.1038/s41598-020-63732-y

**Published:** 2020-04-24

**Authors:** Zhonglei He, Kangze Liu, Laurence Scally, Eline Manaloto, Sebnem Gunes, Sing Wei Ng, Marcus Maher, Brijesh Tiwari, Hugh J. Byrne, Paula Bourke, Furong Tian, Patrick J. Cullen, James F. Curtin

**Affiliations:** 1 BioPlasma Research Group, School of Food Science and Environmental Health,, Technological University Dublin, Dublin, Ireland; 2 Nanolab, FOCAS Research Institute, Technological University Dublin, Dublin, Ireland; 3 Environmental, Sustainability and Health Research Institutes, Technological University Dublin, Dublin, Ireland; 4 Department of Food Biosciences, Teagasc Food Research Centre, Ashtown, Dublin Ireland; 5 School of Biological Sciences, IGFS, Queens University Belfast, Belfast, United Kingdom; 6 School of Chemical and Biomolecular Engineering, University of Sydney, Sydney, Australia

**Keywords:** Targeted therapies, Nanotechnology in cancer, Endocytosis

## Abstract

Cold atmospheric plasma (CAP) enhances uptake and accumulation of nanoparticles and promotes synergistic cytotoxicity against cancer cells. However, the mechanisms are not well understood. In this study, we investigate the enhanced uptake of theranostic nanomaterials by CAP. Numerical modelling of the uptake of gold nanoparticle into U373MG Glioblastoma multiforme (GBM) cells predicts that CAP may introduce a new uptake route. We demonstrate that cell membrane repair pathways play the main role in this stimulated new uptake route, following non-toxic doses of dielectric barrier discharge CAP. CAP treatment induces cellular membrane damage, mainly via lipid peroxidation as a result of reactive oxygen species (ROS) generation. Membranes rich in peroxidised lipids are then trafficked into cells via membrane repairing endocytosis. We confirm that the enhanced uptake of nanomaterials is clathrin-dependent using chemical inhibitors and silencing of gene expression. Therefore, CAP-stimulated membrane repair increases endocytosis and accelerates the uptake of gold nanoparticles into U373MG cells after CAP treatment. We demonstrate the utility of CAP to model membrane oxidative damage in cells and characterise a previously unreported mechanism of membrane repair to trigger nanomaterial uptake. This knowledge will underpin the development of new delivery strategies for theranostic nanoparticles into cancer cells.

## Introduction

Cold atmospheric plasma (CAP) is increasingly studied in a growing number of clinical trials for cancer treatment^1,2^ and research is ongoing to explore the combination of CAP with other therapies, including nanoparticles, radiotherapy and chemotherapy^3–5^.

Gold nanoparticles (AuNPs) are known to be weakly-toxic to human cells and be readily manufactured and designed for targeting delivery of various therapeutic compounds into cells. Citrate-capped cationic AuNPs may adsorb serum proteins onto their surface and thereby stimulate receptor-mediated endocytosis^6^. Without special surface functionalisation, AuNPs enter cells and become trapped in vesicles^6–8^ or enter the nucleus, depending on their size/shape^9,10^. Meanwhile, AuNPs with functionalised surface chemistries/ligands can directly penetrate the membrane and enter the cytoplasm^11^.

Recently, AuNPs have emerged as a promising reagent, combined with CAP, for anti-cancer therapy^4,12,13^. CAP generates a unique physical and chemical environment, including generating short- and long-lived reactive nitrogen species (RNS, e.g. excited N_2_, N_2_^+^, ONOO^−^ and NO^•^, etc.) and reactive oxygen species (ROS, e.g. ^•^OH, O, ^•^O_2_^−^ and O_3_, etc.), photons as well as heat, pressure gradients, charged particles, and electrostatic and electromagnetic fields, many of which are known to induce biological effects^14–16^. Parallels to this can be found in phagocytes of the immune system. Enzymatic production of RONS along with various hypohalous acids, especially hypochlorites, play a significant role in respiratory bursts, also known as oxidative bursts, which are used in the clearance of tumour cells by phagocytic immune cells including neutrophils, macrophages and monocytes^17^. Anti-cancer cytotoxicity induced by respiratory bursts has been shown to induce spontaneous regression in mouse tumour models^18–20^.

Reactive species can induce a free radical chain reaction in membrane lipids leading to lipid peroxidation, oxidative degradation of the lipids, disruption of membrane function and induced injury and disorder in cells. Peroxidated lipid products can induce further propagation of free radical reactions^21^. Several ROS and RNS generated by CAP can induce cell injures via lipid peroxidation^22^, for instance, hydroxyl radicals (^•^OH) react with various cellular components, including membrane lipid^23^ while superoxide (^•^O_2_^−^) can form peroxynitrite (ONOO^−^), which is able to initiate lipid peroxidation, after reacting with nitric oxide (NO)^23^. RNS, such as NO_2_ and ONOOH, also interact with lipids to form nitrated lipids, which have been demonstrated to play roles in vascular and inflammatory cellular signalling pathways^22^.

Independent application of AuNPs and CAP for cancer therapy has been widely investigated. Recent studies have shown that combination of AuNPs and CAP is emerging as a novel and promising therapeutic approach against malignant tumours, resulting in synergistic anti-cancer effects. Synergistic cytotoxicity has been demonstrated for various AuNPs when combined with CAP against several cancer cell lines including melanoma^4^, breast cancer^12^, glioblastoma^24^, alveolar basal epithelial cancer^25^ and colorectal cancer cells^13^.

In our previous study, we demonstrated CAP in combination with AuNPs shows promising synergistic cytotoxicity to U373MG Glioblastoma multiforme (GBM) cells and ATP-dependent uptake mechanism^26^. Kim *et al*. demonstrated that epidermal growth factor-conjugated AuNPs combined with CAP induced DNA damage and selective apoptosis of A549 human cancer cells after uptake via receptor-mediated endocytosis^25^. Shi *et al*. found that CAP treatment can induce iron-dependent oxidative stress to stimulate fluid-phase endocytosis in in mesothelioma cells^27^.

However, the precise route of uptake requires further investigation. Herein, we demonstrate, for the first time, that synergistic effects of nonfunctionalised AuNPs combined with CAP is via clathrin-mediated endocytosis pathway and is triggered as part of the CAP-induced membrane repair process in GBM cells. Numerical modelling of the uptake of AuNPs, confirmed that CAP treatment stimulated a new uptake route separate from normal cellular processes and in response to lipid peroxidation. Meanwhile, our use of CAP as an alternative novel tool to trigger and understand membrane oxidative damage and repair mechanisms is outlined.

## Results

### Numerical modelling of the uptake of AuNPs by GBM cells

**Figure 1:**
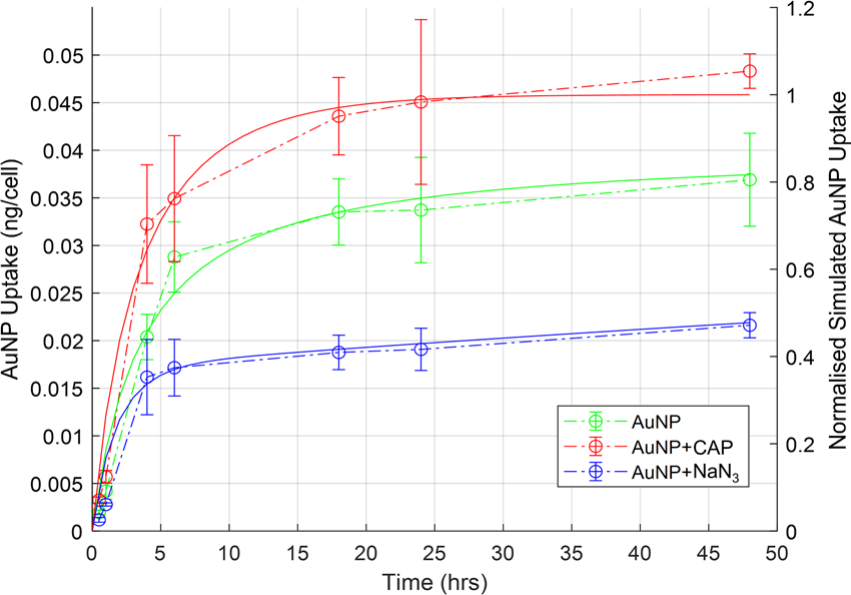
Modelling uptake of AuNPs. Numerical modelling of experimental data from our previous uptake study^26^ (shown with open circles and dashed lines) was carried out for simulated AuNP uptake (green solid line), AuNP uptake quenched by incubation of the cells with NaN3 (blue solid line), AuNP uptake on application of low dose CAP (red solid line). The simulated uptake of AuNP has been normalised to the maximum calculated value.

The accumulation of AuNPs inside U373MG cells was monitored using atomic absorption spectroscopy and the dose response curve of AuNPs with or without CAP treatment has been presented in previous study^26^. The experimental results, reproduced in Fig. 1 (open circles/dashed lines) were further analysed according to a simulated uptake model to better understand the possible mechanism of CAP-stimulated AuNP uptake. Uptake of nanoparticles by cell populations *in vitro* has previously been modelled according to a phenomenological rate equation approach^28–30^, which was extended here to further investigate the role of CAP in AuNP uptake.

The rate of uptake of AuNPs into a cell can be described by the equation:

1 $${{\rm{dN}}}_{{\rm{AuNP}}}/{\rm{dt}}=({\rm{D}}-{{\rm{N}}}_{{\rm{AuNP}}})\ast (1+{{\rm{k}}}_{{\rm{doub}}})\ast ({{\rm{N}}}_{1}\ast {{\rm{R}}}_{1}+{{\rm{N}}}_{2}\ast {{\rm{R}}}_{2}+{{\rm{N}}}_{3}\ast {{\rm{R}}}_{3})$$

where N_AuNP_ is the number of internalised AuNPs, D is the initial dose of AuNPs, (D-N_AuNP_) allows for the depletion of the applied AuNP dose, and k_doub_ is the doubling time of the cells. N_1_/R_1_, N_2_/R_2_and N_3_/R_3_ allow for three different principle uptake pathways, with respective limiting capacities of N and rates R. The first two terms describe independent active and passive uptake mechanisms, respectively, with limiting cellular capacities N_1_(0) = N_1max_ and N_2_(0) = N_2max_, such that:

2 $${{\rm{dN}}}_{1}{\rm{dt}}=-{{\rm{N}}}_{1}\ast ({\rm{D}}-{{\rm{N}}}_{{\rm{AuNP}}})\ast {{\rm{R}}}_{1}$$

3 $${{\rm{dN}}}_{2}{\rm{dt}}=-{{\rm{N}}}_{1}\ast ({\rm{D}}-{{\rm{N}}}_{{\rm{AuNP}}})\ast {{\rm{R}}}_{3}$$

Figure 1 (solid green line) shows the simulated uptake of AuNPs, normalised to the maximum uptake observed for AuNP + CAP, for the case of R_1_ = 3 ×10^−3^ hr^−1^, R_2_ = 2.5 ×10^−5^ hr^−1^, R_3_ = 0, which faithfully reproduces the experimentally observed behaviour. Quenching of the active uptake of AuNPs by NaN_3_ is best simulated by addition of a further term in Eq. 2, such that

4 $${{\rm{dN}}}_{1}{\rm{dt}}=-{{\rm{N}}}_{1}\ast ({\rm{D}}-{{\rm{N}}}_{{\rm{AuNP}}})\ast {{\rm{R}}}_{1}-{{\rm{N}}}_{1}\ast {{\rm{NaN}}}_{3}\ast {R}_{4}$$

where NaN_3_ is the effective dose of sodium azide, and R_4_ allows for the rapid depletion of the active uptake pathway. The experimentally observed uptake was well simulated (solid blue line) by a value of R_4_ = 3 ×10^−5^ hr^−1^, keeping all other rates as before.

In simulating the increased uptake of AuNPs upon CAP treatment, it was noted that the enhancement of a single pathway described by Eqs. (2–4) by CAP treatment, by increasing a single uptake rate, did not faithfully reproduce the experimentally observed behaviour, as the uptakes were limited by the parameters N_1max_ and N_2max_. Rather, faithful reproduction of the observed behaviour required the introduction of independent uptake mechanisms for untreated and CAP treated AuNP uptake, an observation which was critical to the interpretation of the effects of CAP treatment on the cells. Such a pathway can be represented by:

5 $${{\rm{dN}}}_{3}/dt=-{{\rm{N}}}_{3}\ast ({\rm{D}}-{{\rm{N}}}_{{\rm{AuNP}}})\ast {{\rm{R}}}_{3}$$

such that N_3_(0) = N_3max_. Upon the application of CAP, the enhanced uptake was well fitted (solid red line) by R_3_ = 2.5 ×10^−4^ hr^−1^, keeping all other rates as before.

The modelling process indicates that CAP treatment increases the capacity of the U373MG cells to uptake AuNPs by introducing a third uptake route distinct from passive uptake and normal active uptake processes. The modelling parameters employed are detailed in Table 1. Note that the parameters relating to the limiting cell uptake, N_nmax_, were determined by the definition of the dose as 100 mg/mL. Furthermore, the process was one of simulation, rather than a mathematical fitting, so the parameters should be considered within ~10% confidence.

**Table 1:** The modelling parameters employed in Fig. 1.

	N_1max_	N_2max_	N_*3*max_	NaN_3_	R_*1*_ hr^−1^	R_*2*_ hr^−1^	R_*3*_ hr^−1^	R_*4*_ hr^−1^
AuNP	75	150	400	8500	3 × 10^−3^	2.5 × 10^−5^	0	0
AuNP + NaN_*3*_	75	150	400	8500	3 × 10^−3^	2.5 × 10^−5^	0	3 × 10^−5^
AuNP + CAP	75	150	400	8500	3 × 10^−3^	2.5 × 10^−5^	2.5 ×10^−4^	0

### Reactive species generated by CAP treatment

**Figure 2:**
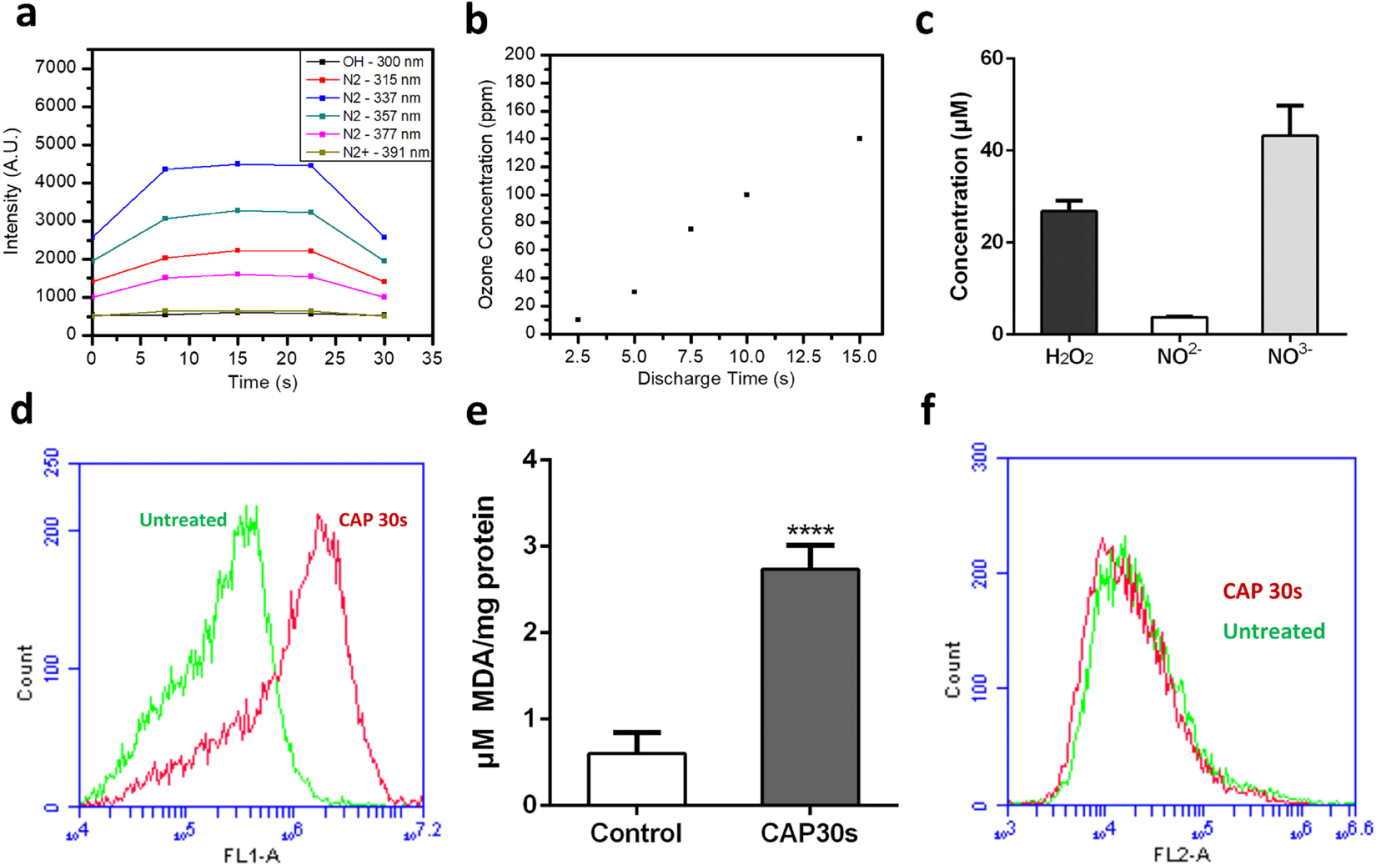
Measurement of reactive species generated by CAP treatment by OES and H_2_DCFDA, TBARS assay and PI staining. (**a**) Emission intensities of excited N_2_ molecules, N_2_^+^ and ^•^OH. (**b**) Concentration of O_3_ measured during CAP treatment. (**c**) The concentrations of hydrogen peroxide, nitrite and nitrate were measured in CAP-treated culture medium. (**d**) Fluorescence level of intracellular oxidised H_2_DCFDA was measured via Flow cytometry, left curve (green, untreated cells), right curve (red, CAP-treated cells). (**e**) U373MG cells were incubated for 24 hours after CAP treatment (0–30 s, 75 kV) and then collected and analysed to detect cellular MDA level using TBARS assay. (**f**) 30 mins after CAP treatment, cells were stained with PI for 5 min, then measured with Flow cytometry.

To identify the possible uptake pathways implicated by the numerical modelling of our data, RONS generation of 30 s, 75 kV CAP treatment was first investigated. Several RONS were measured using optical emission spectroscopy (OES) and Gastec gas detector tubes, including N_2_, N_2_^+^, ^•^OH, and O_3_. OES emission intensities from the N_2_ second positive system (SPS) were measured at 315, 337, 357, and 377 nm, the N_2_^+^ first negative system (FNS) at 391 nm, and ^•^OH at 310 nm were measured. The data demonstrated a relatively constant RONS production throughout the 30 s treatment (Fig. 2a).

The electron energy distribution function (EEDF) of the plasma was also determined. As seen in Supplementary Figure S1, EEDF remained close to a ratio of 7 during CAP treatment, which indicated that the electron energies were distributed more so on the lower end of the energy scale (11–12 eV) than the higher energy levels (18.8 eV). The low variability of the EEDF indicated that the electric field was stable, and that the formation of the reactive species was in a steady state manner. Using Gastec ozone detector tubes, the concentrations of generated O_3_ in the extracted gas were measured post-discharge of CAP treatment (Fig. 2b), revealing significantly increasing levels of O_3_ recorded over the discharge time, which became saturated at the maximum labelled value of O_3_ detection tube after 15 s. High levels of ozone generation may give explanation why no detectable or low emission of NO, O, NO_x_ (NO_2_, NO_3_, N_2_O_2_, N_2_O_3_, and N_2_O_4_), ^•^OH and N_2_^+^ were measured in air using OES^31^ (Supplementary Table S1).

Hydrogen peroxide (H_2_O_2_), nitrite (NO^−^_2_) and nitrate (NO^−^_3_) were measured and detected in culture media (Fig. 2c). Comparing with previous results^32^, CAP treatment of culture media for 30 s generated very low amounts of H_2_O_2_ (~20 μM), NO^2−^(~5 μM), and NO^3−^(~30 μM) which are at least 15-fold and 200-fold lower than the IC_50_ cytotoxicity values we measured previously for U373MG cells (315 µM, >1200 µM and >600 µM respectively) and therefore are essentially non-toxic^33^. Intracellular H_2_O_2_ was significantly elevated 30 minutes after CAP treatment when measured using H_2_DCFDA by flow cytometry (Fig. 2d). Mean fluorescence was observed to significantly increase by 4–5 fold above untreated controls (Supplementary Figure S1).

### CAP treatment induces lipid peroxidation

ROS induces lipid peroxidation of the cell membrane^34,35^. The level of the lipid peroxidation indicator malondialdehyde (MDA), measured using the Thiobarbituric acid reactive substances (TBARS) assay, was significantly higher in U373MG cells 24 h after CAP treatment compared with the control group (Fig. 2e), indicating a high level of lipid peroxidation is induced by CAP treatment. In our previous study, the IC_50_ of CAP treatment (75 kV) was determined to be 74.26 s (95% confidence range of 47.24–116.8 s) for U373MG cells^36^ and we demonstrated that 60 s CAP treatment induces rapid permeabilisation of U373MG cell membranes^37^. Here, we confirm that no significant increase of PI uptake is evident following 30 s CAP treatment compared to the control (Fig. 2f), indicating that membrane integrity was not significantly affected by 30 s CAP treatment and CAP-induced lipid peroxidation. This is in agreement with our previous findings, where 30 s CAP treatment induces very low, non-significant levels of toxicity in U373MG cells 48 hours post treatment (18.52%, SEM = 5.41%)^26^.

**Figure 3:**
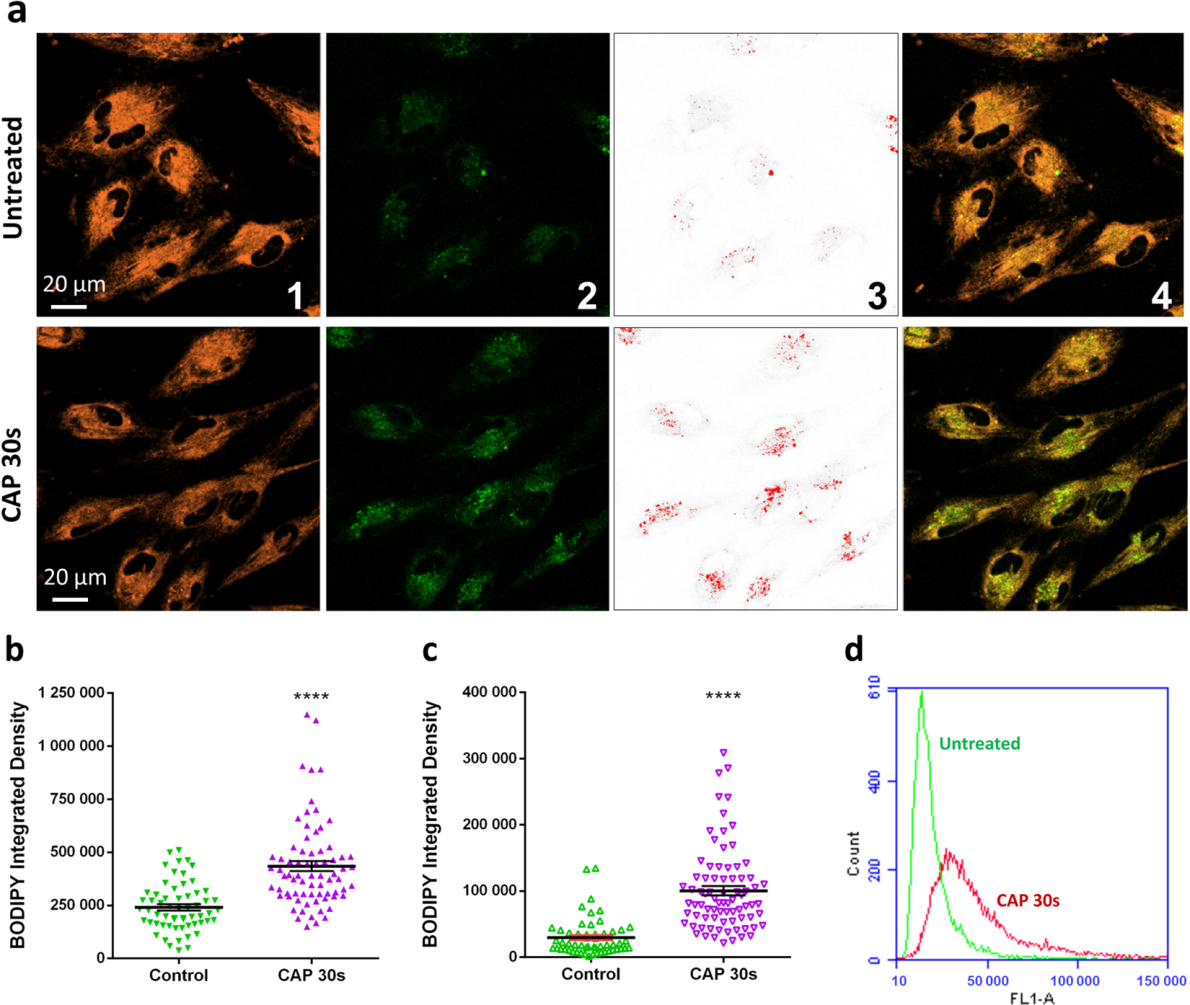
C11-BODIPY (581/591) staining shows lipid peroxidation and membrane trafficking inside the cell to lysosomes. (**a**) Confocal imaging of C11-BODIPY loaded cells, from 1^st^ to 4^th^ panel: Orange, reduced form of C11-BODIPY; Green, oxidised form of C11-BODIPY; Red, vesicle-like structures was highlighted from green fluorescence using high threshold setting in ImageJ; Merged images (orange and green channels). (**b**) The total fluorescent integrated density of oxidised BODIPY was quantified using ImageJ. (**c**) With high threshold setting, the fluorescence integrated density of oxidised BODIPY was quantified with a focus on green fluorescent vesicle-like structures. The statistical significance (**b, c**) assessed by unpaired T-test. (*P < 0.05, **P < 0.01, ***P < 0.001, ****p < 0.0001), n ≥ 50, see Supplementary Table S2 for original data. (**d**) Fluorescence level of intracellular oxidised BODIPY was measured via Flow cytometry, left curve (green, untreated cells), right curve (red, CAP-treated cells).

CAP induced lipid peroxidation was further analysed and visualised using the lipid peroxidation fluorescent sensor C11-BODIPY (581/591). C11-BODIPY (581/591) is a lipophilic fluorescent dye that remains membrane bound, can react with various RONS and oxidation leads to a shift of the emission peak from around 590 nm (Orange) to around 510 nm (Green)^38^. To determine the level of lipid peroxidation, more than 60 cells were analysed using ImageJ software for each group. A significantly stronger emission of the oxidised green fluorescence was observed in CAP-treated cells compared to the untreated cells (****p < 0.0001, Fig. 3a, 2^nd^ panel and Fig. 3b), which was accompanied by a significant decrease in the non-oxidised orange fluorescence (Fig. 3a, 1^st^ panel and Supplementary Figure S2). Furthermore, Fig. 3c demonstrated a 3-fold significantly greater level of the oxidised green fluorescence in CAP-treated cells (****p < 0.0001) with a focus on green fluorescent vesicle-like structures (Fig. 3a, 3^rd^ panel), likely caused by concentrated oxidised lipid membranes internalised in endosomes or lysosomes. CAP-induced oxidation of lipid membranes was confirmed using flow cytometry (Fig. 3d). When cells were counterstained with LysoTracker Deep Red (red), examples of colocalization of oxidised membranes and acidic vesicular organelles (such as lysosomes, late endosomes, etc.) were clearly evident in CAP treated cells (Supplementary Figure S3, middle& right).

### Effects of CAP treatment on AuNPs endocytosis

A membrane repair mechanism has been described where cells can quickly remove damaged regions of membranes from the cell surface through rapid endocytosis^39,40^. These impaired membranes can be trafficked into endosomes and finally into lysosomes. We wished to determine whether rapid endocytosis may contribute to the increased uptake of AuNPs and other materials into cells following CAP treatment. To test this hypothesis, CellLight Early Endosomes-RFP, BacMam 2.0 and CellLight Late Endosomes-RFP, BacMam 2.0 were used to further visualise the route of uptake of AuNPs after CAP treatment. Rab5a and Rab7a chimeras tagged with RFP were transfected and expressed inside cells. Following overnight incubation, Rab5a-RFP and Rab7a-RFP specifically tracked early endosomes and late endosomes, respectively^41^. Cells were then incubated with AuNPs for 3 h after CAP treatment. In Supplementary Figure S4, the white arrows identify examples of co-localisation of AuNPs (red) with early (left) and late (right) endosomes (orange). Lysosomes (green) were also counterstained. We have previously demonstrated that AuNPs accumulate in lysosomes 24 hours after 30 s, 75 kV CAP treatment using confocal imaging and 3D-image construction^26^. We demonstrate here that that AuNPs enter CAP-treated U373MG cells mainly through endocytosis and colocalise with Rab5a and Rab7a chimeras corresponding to early and late endosomes respectively, eventually accumulating in lysosomes (Supplementary Figure S4).

**Figure 4:**
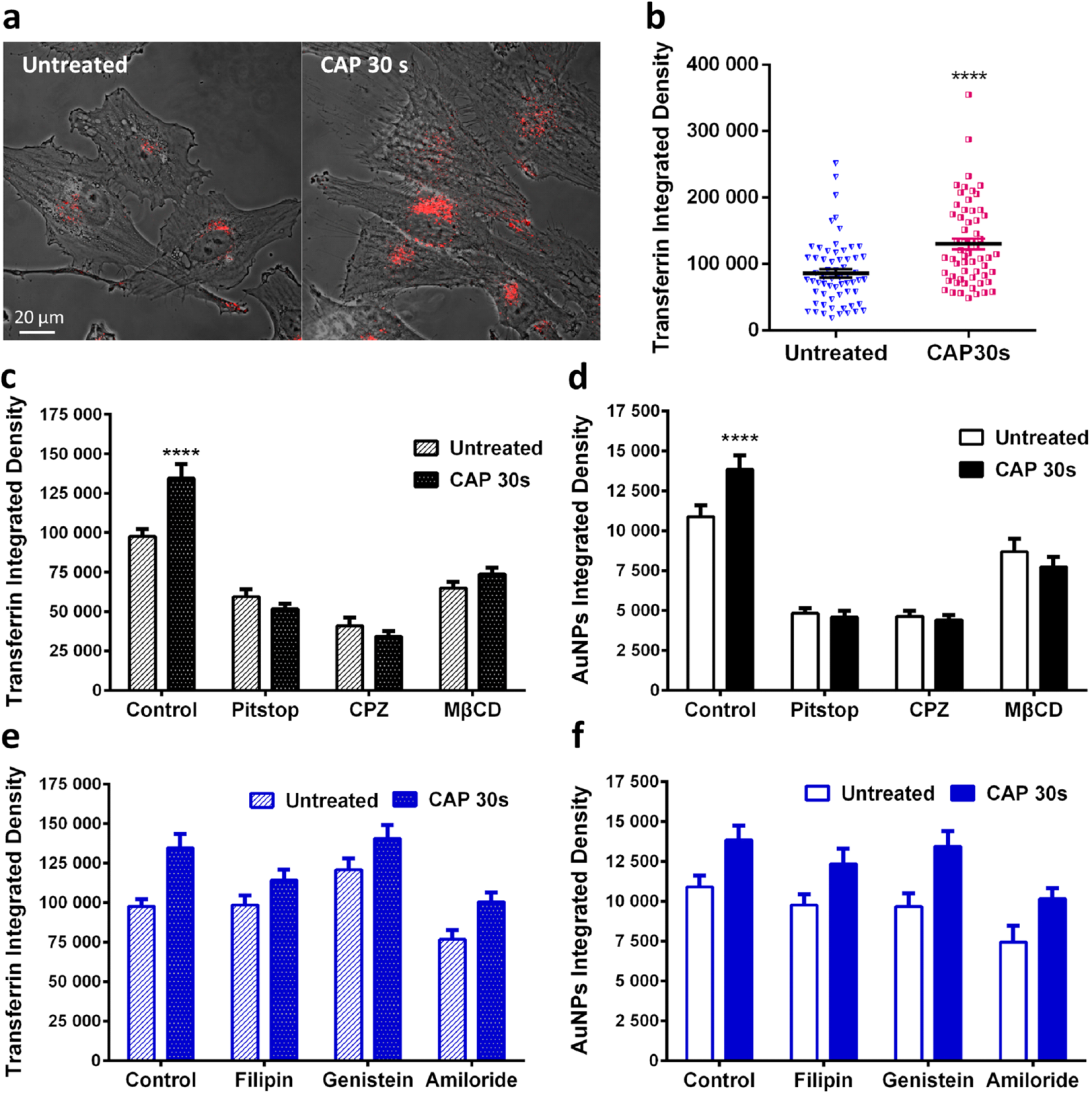
The CAP-induced endocytosis is clathrin-dependent. (**a**) After CAP treatment (0, 30 s), cells were loaded with transferrin-conjugated with Alexa Fluor 546 (red) for 5 min and fixed before observing with a confocal microscope. (**b**) The fluorescence level of transferrin was quantified using ImageJ, see Supplementary Table S3 for original data. (**c**–**f**) After incubation with various inhibitors as indicated, U373MG cells were treated with CAP for 0, 30 s at 75 kV and then loaded with transferrin for 5 min or 100 μg/ml AuNPs for 3 h respectively before observing using confocal microscopy, with the fluorescence integrated densities quantified using ImageJ. The statistical significance in (**b**–**f**) was assessed by one-way ANOVA with Tukey’s multiple comparison post-test (*P < 0.05, **P < 0.01, ***P < 0.001, ****p < 0.0001), n ≥ 50.

Transferrin conjugated with Alexa Fluor 546, is used as an early endosome marker. To investigate the immediate uptake route, CAP treated U373MG cells were incubated with Alexa546-Transferrin for 5 min, then fixed with 4% PFA. As seen from confocal imaging, within 5 mins after CAP treatment, the number of transferrin-containing endosomes was greater compared to the control group (Fig. 4a). Quantification of transferrin uptake confirmed a significant increase of endosomes 5 min after CAP treatment (Fig. 4b, p < 0.0001).

Endocytosis is typically subdivided into four types, including clathrin-mediated endocytosis (CME), caveolae-mediated endocytosis, macropinocytosis and phagocytosis. We used a panel of inhibitors^42^ to delineate the specific endocytic pathway activated by CAP (see Table 2). Clathrin-inhibitors and MβCD-induced cholesterol depletion decreased the number of transferrin labelled endosomes and AuNP-uptake 3 h following CAP treatment (Fig. 4c,d) whereas caveolae-specific inhibitors and an inhibitor of micropinocytosis did not lead to any significant inhibition of Transferrin or AuNP endocytosis in cells 3 h after CAP treatment (Fig. 4e,f). Original data for quantification can be viewed in Supplementary Table S4.

**Table 2:** Inhibitors used to inhibit endocytosis in this research^42^.

AGENT	EFFECT	TARGETING ENDOCYTOSIS
PITSTOP	Small molecular that target and disfunction clathrin^60^.	Clathrin-mediated endocytosis
CPZ	Inhibits Rho GTPase^61^.	Clathrin-mediated endocytosis
MΒCD	Extracts cholesterol from membrane^42^.	CME, caveolae and micropinocytosis
FILIPIN	Interacts with cholesterol^42^.	A few caveolae and cholesterol-dependent mechanisms
GENISTEIN	Inhibitor of few tyrosine kinases^62^	Caveolae pinching
AMILORIDE	Lowers the submembraneous pH and blocks Rac1 and Cdc42 signalling^63^	Micropinocytosis

**Figure 5:**
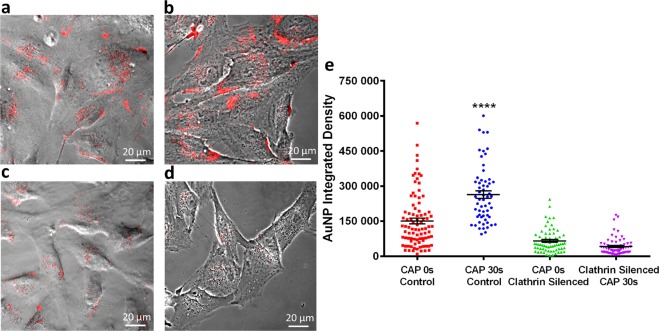
Clathrin silencing inhibits AuNPs uptake and CAP-induced endocytosis. After incubation with esiRNA, targeting expression of clathrin heavy chain, U373MG cells were treated with CAP for 0, 30 s at 75 kV and then loaded with 100 μg/ml AuNPs for 3 h before observation by confocal microscopy, in comparison with unsilenced groups. (**a**) CAP-untreated cells without silencing, (**b**) CAP-treated cells without silencing, (**c**) CAP-untreated cells with silencing of clathrin heavy chain, (**d**) CAP-treated cells with silencing of clathrin heavy chain. AuNPs are identifiable as red fluorescence inside cells. (**e**) The fluorescence level was quantified using ImageJ and presented as integrated density. The statistical significance was assessed by one-way ANOVA with Tukey’s multiple comparison post-test (*P < 0.05, **P < 0.01, ***P < 0.001, ****p < 0.0001), n ≥ 50.

To further confirm that clathrin-mediated endocytosis played the main role in CAP-accelerated cellular uptake, MISSION Endoribonuclease-prepared siRNA (esiRNA) against human Clathrin heavy chain 1 (CLTC) was used to disrupting endocytosis mediated by clathrin coated pit formation. AuNPs uptake was visualised in clathrin-silenced cells and controls treated with or without CAP using confocal microscopy (Fig. 5a–d). AuNP uptake was significantly lower in clathrin-silenced cells and no increase in AuNP uptake was observed 3 h after CAP treatment (p < 0.0001, Fig. 5e and see Supplementary Table S5 for original data). Together, our data confirms that clathrin-mediated endocytosis played an important role in AuNP uptake, and accelerated endocytosis following CAP treatment was clathrin dependent.

## Discussion

The cytoplasmic membrane separates and protects the cellular interior from the exterior environment and provides specific and efficient exchange channels for the remaining intercellular balance and cell viability. Therefore, the integrity of the membrane is vital for all cells. Mammalian cells have developed efficient membrane repair mechanisms that can recover and reseal an injured cytoplasm membrane quickly to retain cell viability. Although investigations of the precise membrane repairing mechanisms have been limited, four possible mechanisms, including patch, tension reduction and exocytosis/endocytosis and budding repair mechanisms have been proposed^40^. The study of cytoplasmic membrane repair usually employs bacterial pore-forming toxins, such as Streptolysin O, to create mechanical injuries on membranes^40^. Meanwhile, lipid peroxidation is a complex process that damages cellular membrane structure and function, which is believed to link to numerous human diseases and aging, including Alzheimer diseases, dementia, Huntington, Parkinson, and traumatic injuries, under presence of oxidative stress^43,44^. Many studies have shown that lipid peroxidation have various significant effects to cellular membranes, such as increased membrane permeability^45–47^, alteration of the lipid order and membrane fluidity^46,48–50^ and activity change of membrane proteins^50–53^. However, there remains a paucity of literature identifying mechanisms of lipid peroxidation-related membrane damage repair, and the mechanisms of oxidised membrane repair remain unknown.

CAP is known to generate reactive species and thereby cause lipid peroxidation of cells. In this research, we explored CAP-induced lipid peroxidation using a low, relatively non-toxic dose of CAP and studied the possible mechanisms of accelerated cellular uptake of AuNPs following CAP-induced oxidative membrane damage.

As seen in Fig. 1, the uptake and accumulation of AuNPs into U373MG cells was first modelled. To faithfully reproduce the experimentally observed results in our mathematical model, a new independent uptake rate was necessary in the model (Eq. 5). This numerical model indicates that CAP treatment may introduce a new uptake route. Following a detailed experimental analysis, we can confirm this occurs and is due to a CAP-triggered, membrane repairing, clathrin-dependent, pathway of endocytosis.

As seen in Fig. 2, we observed a relatively high level of reactive species generated in air during CAP treatment such as ^•^OH, ONOO^−^ and O_3_. This was also evident in media, cells, and ultimately in lipid membranes with the detection of RONS including hydrogen peroxide, nitrite and nitrate and detection of peroxidated lipids and by-products. Interestingly, the low doses of CAP treatment used generate non-toxic levels of RONS but still are induce significant lipid peroxidation.

CAP treatment has been shown to alter membrane structures, which may be partly due to the reactive species-caused lipid peroxidation^24,37,54,55^. However, in our study, cell membranes remains PI impermeable and therefore intact after exposing to 75 kV CAP for 30 s. Interestingly, not only did we observe a significant increase in oxidised lipids using C11-BODIPY following CAP treatment, but there was also a clear and significant increase in vesicle-like structures with high levels of oxidised C11-BODIPY, suggesting that peroxidised membrane lipids were trafficked inside cells via endocytosis (Fig. 3a). An immediate (within 5 minutes) increase in endosomes was also confirmed using transferrin conjugated with Alexa Fluor 546.

Therefore, we propose that low, sub-toxic doses of CAP can cause cytoplasmic membrane oxidation which triggers a rapid membrane repair system. The increased endocytosis induced by membrane repair is initiated within 5 minutes and accelerates the uptake of AuNPs into U373MG cells.

To further explore the route of uptake, AuNPs were tracked in cells counterstained with Rab5a-RFP, Rab7a-RFP, and LysoTracker Green DND-26. Co-localisation of AuNPs with early and late endosomes and lysosomes were observed. AuNPs uptake displayed a tendency to enter the periphery region of the cells through endocytosis, then gather at the central zone of the cells via endosome trafficking into lysosomes. This demonstrates that CAP treatment of U373MG cells triggers uptake and trafficking of AuNPs through early endosomes into late endosomes and ultimately accumulation lysosomes.

Our inhibitor studies and RNA interference studies demonstrate a primary role for clathrin-dependent endocytosis of AuNPs, and not other routes of endocytosis in CAP-triggered uptake. We compared uptake of AuNPs with Transferrin, an essential iron-binding protein that facilitate iron-uptake in cells via transferrin receptors. Uptake of Transferrin is well characterised and involves Clathrin-mediated, receptor dependent endocytosis^56^. Interestingly, Shi *et al*. found that CAP treatment decreased transferrin receptor-1 but could induce iron-dependent oxidative stress to stimulate significant increase in fluid-phase endocytosis, lysosome biogenesis and autophagy in mesothelioma cells^27^. In contrast to this, as seen in Fig. 4a,b, our data demonstrates that the uptake of transferrin was significantly increased just 5 min after CAP treatment, which may due to the diversity of cell lines or CAP generation devices. Similar to AuNPs, formation of transferrin-trafficking endosomes was inhibited in both groups with 0 and 30 s CAP treatment, after exposure to specific/non-specific clathrin inhibitors, including pitstop, CPZ and MβCD. Tracking the accumulation of AuNPs demonstrates the long-term CAP-triggered uptake via a single, clathrin-dependent endocytosis route are retained for at least 24 hours post CAP treatment. Jinno M *et al*. demonstrated in a CAP-induced gene transfection model using mouse fibroblasts that a clathrin specific inhibitor showed 60% decrease in the gene transfection efficiency and that both clathrin-dependent endocytosis and electroporation played a role in enhanced transfection efficiency^57^. The work of Jinno M *et al*. supports our investigation that clathrin may play key roles in CAP-induced cellular uptake mechanisms in response to different materials.

Our data indicate that oxidised lipids can be seen to colocalise with endosomes and lysosomes demonstrating the intracellular sites where the repair of peroxidised lipid membranes is taking place. Further investigation of these mechanisms and sites of membrane repair following CAP-stimulated oxidative damage is ongoing, to identify novel therapeutic targets that can augment CAP cytotoxicity and to fully understand the cellular mechanisms governing oxidative membrane repair.

In summary, we report that the enhanced uptake of AuNPs induced by CAP can be as a result of ROS-caused lipid peroxidation, leading to rapid plasma membrane repair via clathrin-dependent endocytosis. This contributes to our understanding of the cellular effects induced by CAP, especially membrane damage and endocytosis activation, which can be employed for efficient uptake of nanomaterials and pharmaceuticals into cells when combining CAP with cancer therapies. This mechanism of RONS-induced endocytosis will also be of relevance to researchers optimizing other cancer therapies that induce an increase in extracellular RONS.

## Methods

### Cell culture and gold nanoparticle treatment

U373MG-CD14, human brain glioblastoma cancer cells (Obtained from Dr Michael Carty, Trinity College Dublin) were cultured in DMEM-high glucose medium (Merck, Arklow, Ireland) supplemented with 10% FBS (Merck) and maintained in a 37 °C incubator within a humidified 5% (v/v) CO_2_ atmosphere. Gold nanoparticles were synthesised by trisodium citrate reduction of auric acid. 20 nm sphere citrate-capped AuNPs were used to treat cells whose properties were determined in a previous study^26^. The gold colloid was concentrated to 2500 μg/ml then diluted in culture medium to 100 μg/ml.

### CAP configuration and treatment

The current research uses an experimental atmospheric dielectric barrier discharge (DBD) plasma reactor, which has been described and characterised in detail^36,58^. Unless otherwise stated, all U373MG cells were treated within containers, which were placed in between two electrodes, at a voltage level of 75 kV for 30 s. The culture medium was removed prior to CAP treatment then replaced with fresh culture medium afterwards.

### H_2_DCFDA assay and optical emission spectroscopy (OES) and ozone measurement

H_2_DCFDA (Thermo Fisher Scientific, Ballycoolin, Ireland) was used to detect ROS induced by CAP treatment. U373MG cells were seeded into the TC dish 35 standard (Sarstedt, Belfast, UK) at a density of 2×10^5^ cells/ml and incubated overnight to allow adherence. After washing twice with PBS, cells were incubated with 25 μM H_2_DCFDA in serum-free medium for 30 min at 37 °C. Cells were then washed with PBS twice, culture medium once and treated with CAP at 75 kV for 30 s. The fluorescence of H_2_DCFDA was measured using flow cytometry 30 minutes later.

Optical emission spectroscopy was carried out using an Edmund Optics CCD spectrometer with a spectral resolution of between 0.6 nm to 1.8 nm. The spectra was measured using BWSpec^TM^ software with a spectral range between 200 and 850 nm and was acquired every 7.5 s with an integration time of 1500 ms. Total relative intensity of each emission line was calculated using the integral of the area under each peak. EEDF was calculated using a line ratio method (N_2_ at 337 nm and N_2_^+^ at 391 nm)^59^. O_3_ was sampled using a standard Gastec sampling pump in conjunction with a Gastec detection tubes immediately after plasma discharge had ceased.

### Measurement of hydrogen peroxide, nitrite and nitrate concentrations

The concentrations of hydrogen peroxide, nitrite and nitrate were quantitatively measured in CAP-treated culture medium without phenol red. Concentrations of hydrogen peroxide, nitrite and nitrate were determined employing the TiSO_4_ assay, Griess reagent and 2,6-dimethylphenol assay, respectively. The quantitative methods have been described in detail elsewhere^33^. To eliminate the effect of the culture medium on photometrical measurements, the results of CAP-treated groups were standardised with untreated culture medium.

### Lipid peroxidation

For TBARS assay, thiobarbituric acid (TBA), trichloroacetic acid, MDA were purchased from Merck. U373MG cells were seeded into TC Dish 150 (Sarstedt) at a density of 1×10^5^ cells/ml and incubated until confluence. After CAP treatment, cells were further incubated for 24 h, and collected by trypsinisation, centrifuged (100 g for 5 min), and homogenized by sonication. 100 μl homogenate was mixed with 200 μl ice cold 10% trichloroacetic acid and incubated on ice for 15 min to precipitate protein, then centrifuged (2200 g for 15 min at 4 °C). 200 μl of each supernatant was then mixed with 200 μl 0.67% (w/v) TBA and incubate at 100 °C for 10 min. After cooling, samples were measured at 532 nm for MDA.

Lipid peroxidation sensor, C11-BODIPY (581/591) (Thermo Fisher Scientific) was used for *in-situ* detection and localization of the lipid peroxidation induced by CAP treatment. Cells were incubated in fresh culture medium containing 5 μM of the probe at 37 °C for 30 min in advance. Then the cells were washed with PBS twice and culture medium once. After CAP treatment, cells were further incubated with fresh medium for 30 min at 37 °C, and observed using flow cytometry and confocal microscope as described later.

### Flow cytometry

BD Accuri C6 Plus flow cytometry (BD Bioscience, Allschwil, Switzerland) was used in this study. Cells were loaded with C11-BODIPY and treated with CAP as described above (Lipid peroxidation). To prepare aliquots, all floating and attaching cells were collected by trypsinisation and then washed twice with PBS. For the measurement, a 488 nm laser was used for excitation, and 10,000 gated events were collected. Green fluorescence (oxidised dye) and red fluorescence (non-oxidised dye) was measured using an FL1 standard filter (533/30 nm) and FL2 standard filter (585/40 nm), respectively.

For Propidium iodide (PI) staining, cells were exposed to CAP 75 kV for 30 s and incubated at 37 °C for 30 minutes afterwards, then collected by trypsinisation, resuspended into 1 ml PBS. Resuspended cells were stained with 1 µg/ml PI for 5 minutes. The fluorescence of PI was then measured at FL2 (585/40 nm) standard filter.

#### Inhibitor studies

To inhibit various endocytic pathways, cells were pre-incubated with Pitstop (12.5 μM, 5 min) chlorpromazine (10 μg/ml, 10 min), filipin (5 μg/ml, 30 min), genistein (200 μM, 30 min), amiloride (50 μM, 30 min) and methyl-β-cyclodextrin (10 mM, 30 min) in culture medium for the time indicated, at 37 °C. After inhibiting treatment, the culture medium was removed during CAP treatment, prewarmed fresh culture medium containing 100 μg/ml AuNPs was then added immediately to the dishes and incubated for 3 h before observing using a Zeiss LSM 510 confocal laser scanning microscope.

Transferrin conjugated with Alexa Fluor 546 was used to determine the change of early endosomes induced by CAP combining various endocytosis inhibitors. After the inhibiting and CAP treatments indicated above, the cells were incubated in prewarmed fresh medium for 0 or 3 h, then incubated with 25 μg/ml transferrin in medium for 5 min. Afterwards, cells were fixed with 4% PFA and then observed using confocal microscopy. The details of the confocal microscope are described in following section.

### Clathrin silencing

MISSION esiRNA (human CLTC) and MISSION siRNA transfection reagents were purchase from Merck. 50,000 U373MG cells were seeded into each 35 mm glass-bottom dishes (Greiner Bio-One, Lörrach, Germany) and incubated overnight. 1.2 ul esiRNA stock was mixed with 20 ul of transfection reagent in 400 μl serum-free medium and incubated for 15 minutes at room temperature. In glass-bottom dishes, previous medium was replaced with 1 ml of prewarmed fresh medium with serum. The siRNA/transfection reagent solution was then added onto the cells and homogenized to final volume of 1.2 ml. Afterwards, U373MG cells were incubated at 37 °C for 24 h and then incubated with fresh medium containing 100 μg/ml AuNPs for 3 h before observing using confocal microscopy.

### Endocytosis tracking and cell imaging

Early endosomes, late endosomes, lysosomes were demonstrated using the CellLight Early Endosomes-RFP, BacMam 2.0, the CellLight Late Endosomes-RFP, BacMam 2.0 and the LysoTracker Green DND-26, respectively (Thermo Fisher Scientific). 35 mm glass-bottom dishes (Greiner Bio-One) were used as containers for confocal imaging. For early and late endosome marker, 2 μl of BacMan 2.0 reagent per 10,000 cells was added in fresh medium and incubated with cells at 37 °C for 16 h. Then the cells were treated with CAP for 30 s and incubated with fresh medium containing 100 μg/ml AuNPs for 3 at 37 °C. Before observing under a Zeiss LSM 510 confocal laser scanning microscope, cells were washed twice with PBS and incubated with fresh medium containing 50 nM LysoTracker for 5 min at 37 °C. The corresponding filter settings were as follows. AuNPs, excitation 633 nm, reflection 649–799 nm; Transferrin conjugated with Alexa Fluor 546, excitation 568 nm, emission 580–630 nm; CellLight Early Endosomes-RFP, BacMam 2.0, excitation 568 nm, emission 580–630 nm; CellLight Late Endosomes-RFP, BacMam 2.0, excitation 568 nm, emission 580–630 nm; LysoTracker Green DND-26, excitation 488 nm, emission 505–530 nm; C11-BODIPY (581/591), excitation 1: 488 nm, emission 1: 500–560 nm; excitation 2: 568 nm, emission 2: 560–620 nm. Plan-Apochromat 63×/1.4 Oil Ph3 was used as objective for all samples. The integrated density of fluorescence in the confocal images was quantified using ImageJ software. The confocal images were unscaled, the quantified integrated density being the sum of the pixel values in the selection, which is the selected fluorescent area.

### Statistical analysis

At least triplicate independent tests were carried out for each data point, unless indicated otherwise. Error bars of all figures are presented using the standard error of the mean (S.E.M). Prism 6 (GraphPad Software) was used to carry out curve fitting and statistical analysis. Two-tailed P values were used and the Alpha for all experiments is 0.05. The significance between data points was verified using one-way ANOVA and two-way ANOVA with Tukey’s multiple comparison post-test, as indicated in figures (*P < 0.05, **P < 0.01, ***P < 0.001, ****p < 0.0001).

## Supplementary information

Supplementary Information.

## Data Availability

All data generated or analysed during this study are included in this published article.
