# Supplementary material for: Cold Atmospheric Plasma Stimulates Clathrin-Dependent Endocytosis to Repair Oxidised Membrane and Enhance Uptake of Nanomaterial in Glioblastoma Multiforme Cells
Source: Sci Rep. 2020 Apr 24;10:6985. doi: 10.1038/s41598-020-63732-y (PMC7181794; doi:10.1038/s41598-020-63732-y)
Supplement: Supplementary file 1 — Supplementary Information. [file 41598_2020_63732_MOESM1_ESM.pdf]

TITLE:

Supplementary Information:

Cold Atmospheric Plasma Stimulates Clathrin-Dependent Endocytosis to Repair Oxidised Membrane and Enhance Uptake of Nanomaterial in Glioblastoma Multiforme Cells

Zhonglei He[1,2,3], Kangze Liu[1,2,3*], Laurence Scally[1*], Eline Manaloto[1,2], Sebnem Gunes[1,2], Sing Wei Ng[1,3], Marcus Maher[2], Brijesh Tiwari[4], Hugh J Byrne[2], Paula Bourke[1,3,5], Furong Tian[1,2,3], Patrick J Cullen[1,6], and James F Curtin[1,2,3]

[1]BioPlasma Research Group, School of Food Science and Environmental Health, Technological University Dublin, Dublin, Ireland; [2]Nanolab, FOCAS Research Institute, Technological University Dublin, Dublin, Ireland; and [3]Environmental, Sustainability and Health Research Institutes, Technological University Dublin, Dublin, Ireland; [4]Department of Food Biosciences, Teagasc Food Research Centre, Ashtown, Dublin, Ireland; [5]School of Biological Sciences, IGFS, Queens University Belfast, United Kingdom; [6]School of Chemical and Biomolecular Engineering, University of Sydney, Australia

*These authors contributed equally to this work.

Corresponding author

Correspondence to James Curtin (james.curtin@dit.ie).

Supplementary Materials

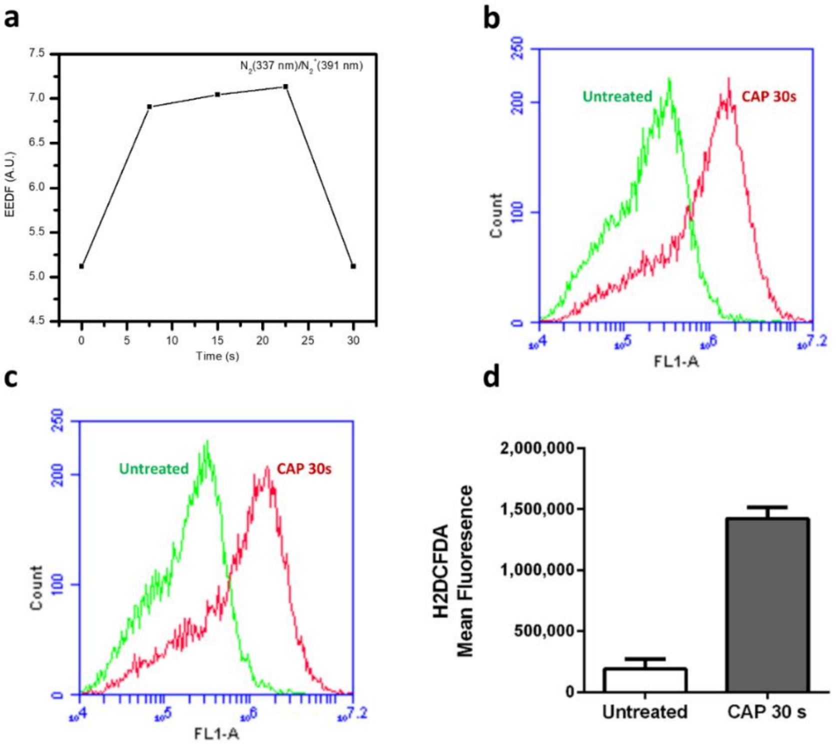

Supplementary Figure S1. *(a) Electro energy distribution function (EEDF) of CAP*; *Fluorescence level of intracellular oxidised H_2DCFDA was measured via Flow cytometry, two replicas (b, c) and the analysed average of mean FL1-A value (d).*

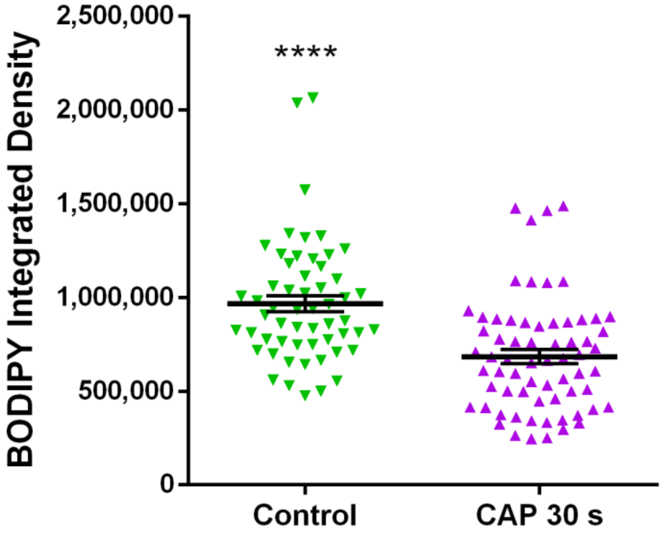

Supplementary Figure S2. *The fluorescent integrated density of non-oxidised BODIPY was quantified using ImageJ. The statistical significance was assessed by one-way ANOVA with Tukey's multiple comparison post-test (*P<0.05, **P<0.01, ***P<0.001, ****p<0.0001), n≥50, see Supplementary Table S2 for original data*

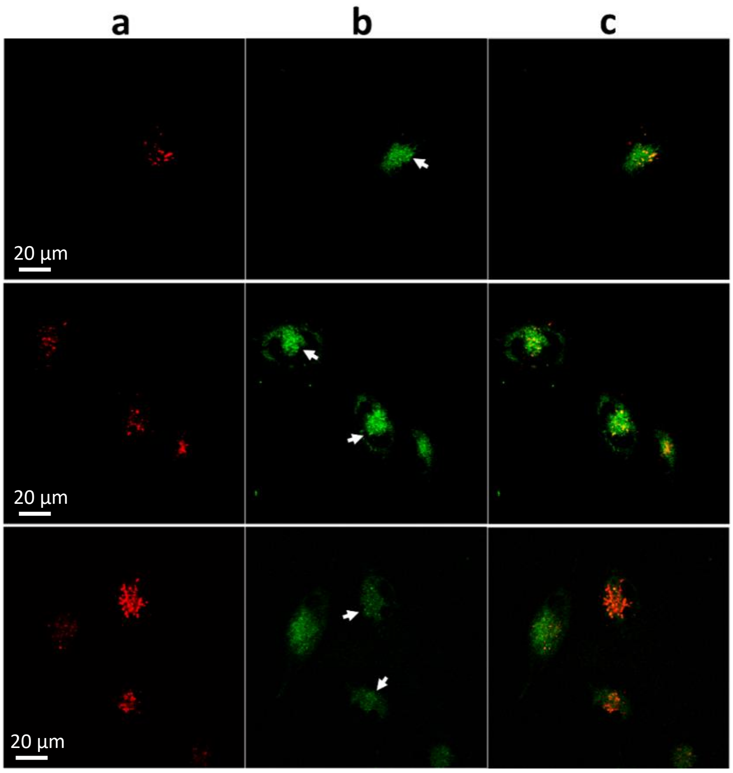

Supplementary Figure S3. *The U373MG cells were loaded with C11-BODIPY (green), then co-stained with and LysoTracker™ Deep Red (red) after CAP treatment (30 s, 75 kV) and observed under confocal microscope, three images are presented in the figure. White arrows point out examples of co-localisation between oxidised C11-BODIPY and lysosomes.*

Supplementary Table S1:

The intensity of O_3 production may explain why there was no detectable emission of NO, O, NO_x and low intensities of $^{\bullet}OH$ and N_2^+ in Figure 2a, as the free electrons were likely quenched before reaching higher energetic states by the interaction and formation of O_3. Reaction mechanisms (1-4) can further explain how the formation of NO, NO_x, and O was hindered. In these reactions, M is a third body atom or molecule that, in this case, may be N_2^* or O_2.

$\mathbf{O + O_2 + M^* \rightarrow O_3 + M}$	**(1)**
$\mathbf{M^* + NO \rightarrow M + N + O}$	**(2)**
$\mathbf{O + H_2O \rightarrow 2\ ^{\bullet}OH}$	**(3)**
$\mathbf{2N + M \rightarrow N_2 + M}$	**(4)**

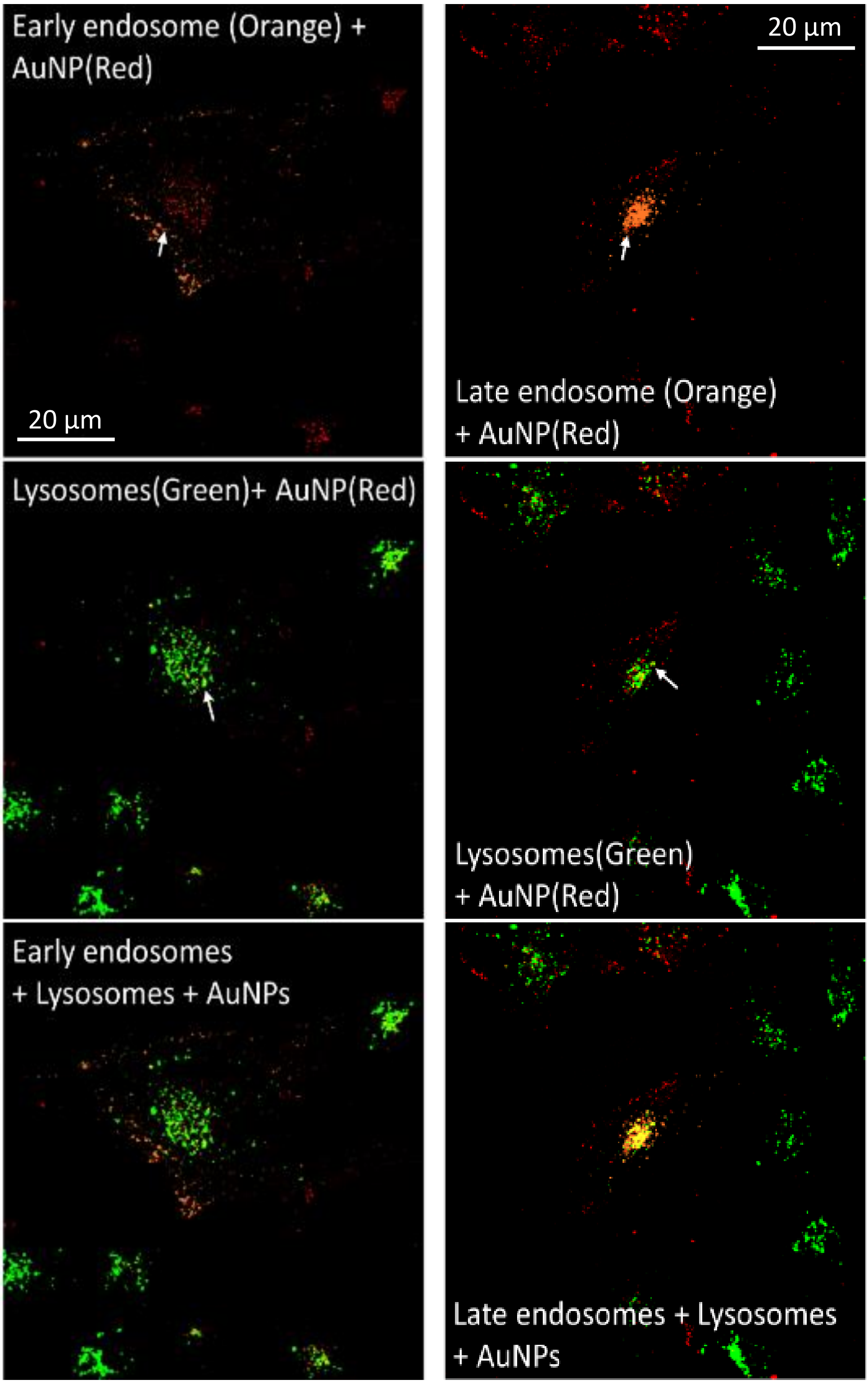

Supplementary Figure S4. Uptake and subcellular localization of AuNPs observed by confocal microscopy. *After CAP treatment (0, 30 s), U373MG cells were incubated with 100 μg/ml AuNPs for 3 h. Early, late endosomes and lysosomes were stained using CellLight™ Early/Late Endosomes-RFP (Marked as orange channel in images) and LysoTracker™ Green DND-26, respectively. The far-red emission of AuNPs (Red) was excited using 633 nm laser.*

Supplementary Table S2.

Integrated Density of BODIPY			
**Control. Non-oxidised**	**CAP 30s. Non-oxidised**	**Control. Oxidised**	**CAP 30s. Oxidised**
825441	764257	232430	417629
998949	760580	310618	342874
1060462	665879	327636	495228
859260	1089862	179182	477016
530275	765581	307326	426428
829356	360915	176954	448261
665668	551518	499637	293395
1278050	264406	510195	325466
2038367	596517	356545	273752
936308	760052	292490	464481
1259645	332197	407417	167146
1573896	866259	281113	394278
1019896	499450	227051	302926
963641	296252	181733	221856
862036	846493	189916	454239
812204	881837	248568	374735
1113481	861837	300329	373363
748271	778087	273441	505987
1165023	524382	211968	292939
764072	867958	282522	388750
1232175	508610	317893	597744
1007005	595270	161873	295272
982070	610424	155628	306654
1320705	445595	458594	390399
946150	1463426	399135	890896
1342500	708723	462811	741382
653886	888079	196388	524934
499775	373856	190853	306468
875556	1477152	153302	390161
1037540	653049	163432	651081
906223	1078557	142877	187065
1097569	675892	206789	906811
1207059	402444	37404	312273
718964	1083730	109317	617780
559771	930197	236604	468648
475472	898191	142135	296259
747381	331716	270552	389688
807312	532198	82436	195602
707968	817839	84652	506283
840742	414865	168778	451699
777642	749632	364207	303635
1022745	879071	100734	431292
1228769	1084756	114519	701205
2064986	412708	49002	444981
697882	884861	273032	223330
1220345	566726	58729	423576
775782	667011	169249	569403
1182171	895365	184585	660069
812884	497905	198073	227918
940440	603690	154684	478087
555395	684168	441372	254274
1327438	371186	274210	362961
718786	502118	245082	398478
642920	415450	228162	325251
1050097	1489285	437520	346672

834466	250668	350830	479690
	247114	199513	1122291
	460969	184087	191401
	345379	260743	335414
	325823	234688	346672
	342280		303883
	694899		306629
	607436		1149608
	821919		150280
	1413574		456893
	729336		283002
			269830
			234559
			524097
			372300
			626520
			889340
			691489

Integrated Density of BODIPY with High Threshold	
**Control. Oxidised**	**CAP 30s. Oxidised**
19018	34784
4765	40598
16250	46050
7714	59301
4375	47707
88177	31247
13826	285541
13985	136701
20766	119089
44683	190134
21404	40796
38923	81647
134601	308360
49402	134591
133104	106744
19026	78642
6092	136430
8353	169067
29580	217020
17094	37454
35776	24779
13745	52852
76947	29677
1924	81249
13232	117778
12490	68022
45683	190817
50730	88448
25208	164970
12313	118069

11361	44102
6732	98463
7489	51073
28732	241221
70660	30053
12378	101954
14617	68583
22167	60325
14598	198866
35108	77078
55412	177196
34319	105262
67507	68627
11005	99365
37081	86536
41038	145387
18167	118557
19262	93622
35329	109825
14752	80553
25135	21300
41692	97853
9707	71932
15904	42466
8189	120579
	71679
	136028
	59030
	32224
	43704
	81999
	81288
	78779
	54076
	95249
	42523
	65630
	242304
	142045
	59181
	66497
	278031
	118417
	119211
	78988
	98313

	62779
	72525
	96661

Supplementary Table S3.

Integrated Density of Transferrin	
**Untreated**	**CAP30s**
110807	57027
66999	112562
125539	86679
25907	97764
18663	210017
36431	151799
55003	82789
58136	180386
108838	219322
203364	120825
38237	109507
106023	181812
153065	232475
46973	103867
231021	165690
126260	189547
58261	145942
53516	216243
28616	175271
169818	79758
119425	197285
69592	173238
24623	207385
126382	137586
109035	103140
251395	55164
90739	287846
76501	49045
76803	75313
71061	76832
70167	89317
71700	116572
29972	81825
28606	205643
87132	134411
33269	101051
95125	355132
80060	57270
64769	73021
106737	108349
74147	88346
79054	56330
25151	115220
40603	109490
72073	124859
124198	218535
77852	134532
103302	71167
109488	71050
58064	60830

75705	58081
27911	63689
86282	181942
117192	170011
32705	89790
120661	71784
129909	163049
106761	107358
90118	132343
39569	
164849	
84330	

Supplementary Table S4

Integrated Density of Transferrin					
**Untreated**	**CAP30s**	**Pitstop**	**Pitstop+CAP**	**CPZ**	**CPZ+CAP**
108598	295322	64736	100433	38802	56788
43443	108782	38572	31750	24310	83128
77433	251156	47517	76352	39598	45220
94477	84583	25591	100240	294486	19690
86584	65841	35782	71304	68324	13416
98793	91549	39735	28401	51158	9444
102070	163102	100772	44936	53180	16446
129770	156221	40368	22378	72708	29576
112724	81989	30864	12836	68844	5404
60643	207294	74733	17119	131656	7596
70525	80463	76017	26816	72856	4568
110091	86986	75272	97123	62814	21856
139093	145578	91199	43380	119252	2280
98220	63260	29856	83012	26884	7814
133442	69745	115762	17713	51452	129394
143649	110816	105188	49522	28458	117634
75022	123363	43457	54983	71198	30926
123196	134115	50026	18278	74608	77612
70338	228112	7877	59622	26936	68470
55592	140503	69660	29797	3384	88596
71972	121850	99571	77787	7454	30684
168227	158303	62211	74743	34034	75958
93020	121401	89301	78765	19250	95484
129316	70365	98921	47823	15276	103746
110653	146501	86540	32383	49392	9158
88591	129369	84316	42288	15354	4520
122648	82011	80932	52147	19722	25244
75639	98961	76763	42159	51914	17308
131539	112977	188739	101745	54704	50974
157044	76354	30436	28371	41096	16284
163738	94709	46748	63526	36212	5584
78925	51942	34317	91475	23016	19136
111987	177314	59776	41096	19956	7634
125506	94675	35976	56949	17996	30856
125495	129185	57772	69132	31342	84574
120301	127507	56559	99148	19040	30892
105738	90869	90776	41119	56680	9672
48458	119518	32429	59855	24088	12082
68317	113258	58616	47787	42838	37932
36999	247984	18765	73103	18410	4872
72117	277862	67164	27352	9752	61354
89229	184301	72684	37608	22664	29434
45164	167793	38795	41806	21144	36554

77146	317787	49816	72871	63528	27876
88215	213580	12847	87006	15258	69646
91529	72160	56799	66654	18400	59842
80253	60324	26547	49931	64260	23916
63880	97864	15265	46675	20094	14374
120907	156754	56795	29802	18770	79040
86245	125687	23657	64467	27556	61076
			12833	17776	14866
			28102	69252	85876
			38183	19000	15354
			29502	34386	8102
			37891	8592	38704
			61498	11670	34414
			51505	37184	78286
			35668	8968	16564
			24266	21180	71468
			51119	90982	10220
				22680	19342
				42116	16544
				7278	9582
				6646	77040
				59824	30404
				8114	54566
				19838	48010
					7918
					16944
					6710
					7116
					34934
					7930
					67322
					8512
					5804
					8342
					26376
					33834
					2612
					17928
					20240
					9364
					22084
					53872
					38896
					26456
					15434
					43858
					11738

Integrated Density of Transferrin							
MβCD	MβCD+CAP	Filipin	Filipin +CAP	Genistein	Genistein +CAP	Amiloride	Amiloride +CAP
131419	66932	97900	163871	101792	133841	196235	124569
124614	93007	129504	187302	133971	118480	138704	135077
69503	97275	110897	280194	65462	127431	29079	51297
75277	46598	90356	105230	147728	283770	72203	88474
76750	77640	104776	76422	147365	103548	64304	112269
18178	92103	73574	85766	68864	51402	72146	45995
81444	24984	159303	113291	72395	55713	83737	14594
88041	95210	124298	90004	101640	94243	54808	47209
57794	98855	108271	232008	96453	244560	33708	144728
51151	89031	72235	76414	174993	104571	31934	49935
76552	75078	33351	137916	271282	93475	64961	123415
86342	85701	30163	116268	221595	142366	56665	114787
40633	59098	62316	77831	83503	113373	48094	163106
97243	91913	65030	77608	145251	94037	30674	99006
36007	93666	109848	134224	99669	83792	97877	104122
79613	93835	95871	82976	72559	161527	99403	145760
79863	73324	106984	164104	202898	247658	60424	131902
115776	69286	157208	74697	51052	82664	76203	117916
47129	35314	109433	78977	58917	65763	37386	43884
36055	46718	27594	83753	112259	87005	81334	139639
87390	83701	79618	146374	140919	179148	29538	137618
62452	56757	89721	152046	163308	75257	65159	73523
93547	68512	194085	135891	67768	260961	79248	115470
74815	43701	36909	127681	69400	94462	34570	38530
65652	38500	50967	92144	52293	252478	101412	214459
90255	46948	159055	91692	74286	71411	40862	55017
71303	102306	165479	64158	119649	159244	30034	72765
50020	90653	48369	143926	121836	167913	78864	82769
76361	105447	39814	83297	157275	103238	21499	145664
61373	48421	82143	93936	100709	117632	87521	103887
72042	14139	117174	100813	268448	56085	32872	96235
89546	27184	96452	101386	96458	122851	86774	107471
94135	58249	61675	144732	200374	244874	108724	73740
70875	29039	152249	50729	133156	172584	99005	138795
46285	118411	51630	72558	123251	93479	91400	109758
74820	60569	178335	116879	66756	99088	162496	97441
64908	69129	58091	92915	148409	156027	59966	55005
49964	19395	109739	150147	156027	139595	83504	122417
25266	97511	122864	126267	116189	127740	79816	96430
12030	99651	62501	88480	131616	188948	70233	105109
68275	94682	115067	70907	145548	247489	54977	82412
24230	53849	89403	87317	64925	129827	20755	137153
48315	77480	144868	91462	99657	129957	87959	62907
56293	124595	133432	158029	129320	108170	113851	62485
46775	125449	143229		126041	113664	9751	139596

53149	67036	50177		113392	224130	195704	
61754	101836	60733		69885	249655	205460	
18804	127051	147230		81771	112130	71758	
23564	78954	74873		256252	137488	60650	
46598	56597	64963		185763	246213	102646	
		173794		90235	99266	104360	
				87308	88875	72262	
				34485	121148	62646	
				64651	215831	106858	
				116152			
				160153			

Integrated Density of AuNPs					
**Untreated**	**CAP 30s**	**Pitstop**	**Pitstop+CAP**	**CPZ**	**CPZ+CAP**
5516	22261	6083	3792	7990	8366
3581	12306	4177	6099	1863	1957
27847	10762	1746	2099	1486	8966
15965	3480	3011	1812	1434	6266
2647	10667	4032	1975	5801	1452
3198	4966	4808	6106	4778	3468
6305	8758	3805	2532	7212	2421
5788	20948	7747	1728	5556	2566
3919	8903	7417	3160	5631	3101
17942	10360	1322	3273	4089	4259
4457	17401	2229	2863	2949	2871
18099	50518	4509	4671	4297	3558
5395	21642	5231	5348	2607	3562
10992	9222	2962	2937	5111	7809
16843	11587	2971	4733	2967	7133
7621	14788	2567	3325	8772	4191
17892	13311	6843	5923	5787	3747
21501	20808	1608	7813	5110	8074
2455	12197	10176	2810	3289	5436
8796	8593	6170	2574	2880	3060
11688	10932	1970	9692	7444	2601
3500	15539	3286	2373	9698	3402
13317	20754	8646	4150	3923	3123
4841	8846	2766	8823	5399	2857
2679	4831	1331	4191	2188	5444
17626	16624	5692	1857	5285	6896
1669	10846	4185	3482	4443	3207
8186	16627	5613	4884	4856	4577
9202	19013	6779	10185	3336	3296
9836	18567	5571	4395	7300	3899
8877	12700	3958	2648	2034	3728
10929	10548	3951	1977	2889	2222
8565	6980	2132	4485	5657	3424
7736	12991	2702	5126	3539	6041
2356	15723	1284	4665	3242	3298
2851	29901	2677	5481	7771	8405
23594	7900	5960	2430	3372	4964
14115	24781	4984	4714		4473
5866	12827	7586	3752		1527
25137	11509	4864	4573		3776
11108	31897	9875	1779		4807
9864	4429	5750	2711		2822
16203	5919	6359	4560		4205
11111	7653	6231	3132		3023
9532	14639	4619	1628		10408

8395	13590	1388	6677		
11415	8222	4931	12145		
11279	15100	2072	22026		
11099	26498	3939	5527		
13153	14704	3847	2204		
12553	5136	2878	2964		
13217	17050	5958	3948		
17633	9468	6171	7410		
4742	7312	9188	2197		
11052	4848	3643	1465		
15608	13345	1471	5365		
5281	10442	8934	4764		
15270	17738	7551	1713		
12153	10179	2156	2552		
14067	7909	9601	2816		
13005	12894	15586	1916		
6557	10555	7303	6227		
14369	29626	3134	7636		
28194	9385	3070	16947		
4478	13564	6450	2184		
7769	26029	4504	5572		
15100	18355	1676	2971		
14411	8849		4198		
5912	6822		3081		
9159	10362		8244		
10064	12428		6451		
22400	9474		2825		
15100			733		
3145					
5178					
16426					
27029					
8473					
6730					
4345					
9773					

**Integrated Density of AuNPs**							
**Filipin**	**Filipin +CAP**	**Genistein**	**Genistein +CAP**	**MβCD**	**MβCD +CAP**	**Amiloride**	**Amiloride +CAP**
14925	45932	7861	8525	10364	5563	4919	12384
13177	6400	3124	8659	3826	6803	6343	8314
6253	6224	7004	17234	5294	2748	8187	5489
4841	21801	8737	11437	8912	3850	3139	4808
17947	15017	12423	6792	14276	17048	5059	13403
4950	6373	6463	11330	9686	4090	1863	8839
5319	9478	6621	12569	8051	8453	4450	13091
11753	17365	9855	29753	8871	4153	2650	24387
8816	10640	9297	5810	5919	5268	2781	14911
3601	12295	15754	9558	4132	5229	13092	6714
4623	5898	2416	10012	29822	13267	1360	11432
14331	6364	8974	12247	9759	8702	1107	7042
3477	26822	26687	4487	12254	6550	2166	8005
10509	18101	13251	11413	10175	8515	3635	7039
6237	15876	3005	24979	4763	9959	5746	10338
7240	13389	3677	26444	20656	8809	12468	5008
7500	33586	1610	20449	5935	4552	7872	8225
3569	10446	21888	11668	8371	3501	2171	8509
3443	12871	16131	9829	11152	7595	4149	5992

17505	6565	12798	22269	4376	10268	13666	8063
11009	10467	10002	7997	12687	6862	4572	9861
14142	5683	20026	21671	7365	5081	9826	11328
12541	14049	4944	4504	5930	12182	8787	11552
10120	17180	8411	10937	17596	7779	5460	13135
6846	7140	17666	14563	8740	6003	14653	9408
7556	13706	7391	14822	15900	5778	16330	5093
8475	14675	9217	19264	4593	18306	7051	12713
15018	18780	14439	39774	2451	13608	28750	7968
24729	9029	10800	12115	3554	17698	12822	4766
6180	9180	10289	9980	3791	2271	7980	5260
8662	7303	4240	10558	13315	8238	2636	8735
16450	17269	5030	10707	5436	6113	4535	12229
9587	5057	5329	7643	10356	3952	4568	7559
11228	14892	4067	18347	1700	8427	2574	13070
11317	11273	27835	10790	5436	5052	2969	12427
5662	3751	21257	9966	3554	11620	2219	15870
16028	6097	5817	10412	2462	6685	3242	8116
9972	7096	5579	11849	15179	5245	2159	13472
9829	14357	19417	11330	6384	5810	5537	6946
13112	5305	1497	7456	6338		20414	7951
14690	4870	5149	10368	10377		4421	19438
14599	6517	6111	8107	4391		9412	9825
3840	6081	9586	12417	9562		11953	5042
8961	10141	8745	16088			5431	8533
7528	14356	9156	18577			2471	24524
5024	10728	14895	25898			34529	
10471	4837	10299	16529				
4401	12326	10661	9265				
9953	11511	3269	13413				
	8079	3960	7929				
	20982	6973	9668				
	9719	11519	10979				
	6663	6567					
	16067	3955					
	9929						
	26445						
	13543						
	13793						
	12121						
	13008						
	7982						

Supplementary Table S5.

Integrated Density of AuNPs			
**CAP30s Control**	**CAP 0s**	**CAP 0s**	**CAP 30s**
540329	172547	71657	81386
317286	431535	52885	26174
366683	78334	50113	83853
118864	227289	98205	62198
389856	182028	58882	113274
260748	95261	213664	176140
255370	67797	32246	90214
143714	155367	84077	155663
254835	143994	18060	167904
241522	409060	130518	52593
173393	91173	82556	22219
437882	98606	20741	19181

301708	372871	42321	16830
283998	42174	50000	36912
529897	192966	22797	11897
132739	43429	32307	67489
325045	171486	111229	22481
256487	155863	165385	45392
225093	218957	243576	12669
459960	332020	115717	14759
242832	163495	32769	59857
102314	178575	134059	29691
274004	43099	91085	36894
319820	116779	96918	46448
144878	33679	42816	24724
313094	281964	92220	41174
215681	474462	163157	12320
426863	78871	107430	42693
169499	25139	78092	54199
449778	157231	44965	26720
207286	25451	46103	68806
600642	105195	99071	50186
191247	124412	169342	18736
175692	128267	80229	16030
167735	312896	36048	55855
195195	129867	76657	106951
195351	127963	117916	17979
131726	352128	22666	39283
328548	257630	66817	17452
212497	116701	53062	34551
453971	134485	35270	10871
318022	113204	33485	43176
335974	203686	37665	6543
135597	81301	39651	20391
226104	258075	18179	9906
136796	117997	56467	43154
168061	189202	26238	33298
530325	354143	16544	6978
310895	179360	42583	10313
156015	249807	10222	7391
118700	104282	19974	18762
122974	189066	53493	56664
133195	84785	27174	8288
267536	141462	21953	72528
200762	345977	148609	24130
299249	77114	73347	13301
94514	47361	62139	18333
293151	43665	54334	20520
176776	9389	4625	17705
	270307	6653	38519
	31190	46708	28687
	257884	50733	15938
	155736	49224	
	345060	17503	
	43585	15541	
	81386	3876	
	23622		
	75993		
	65180		
	568575		
	318601		
	174400		

	52373		
	23718		
	59279		
	38939		
	75419		
	98606		
	24077		
	75776		
	41710		
	22592		
	70125		
	45832		
	98998		
	114681		
	126842		
	72896		
	161663		
